# Nitroflavone Derivatives Inhibit the Production of Actinorhodin in Streptomyces *coelicolor*

**DOI:** 10.3390/cimb48070744

**Published:** 2026-07-21

**Authors:** Sana Slimene, Michelle David, Daniel Dauzonne, Renato Bensasson, Marie-Joelle Virolle, Fathi Moussa

**Affiliations:** 1 Group MES (Métabolisme Energétique des Streptomyces), Institut de Biologie Intégrative de la Cellule (I2BC, UMR 9198), Commissariat à l’Energie Atomique et aux Energies Alternatives (CEA, CNRS), Université Paris-Saclay, 1 Avenue de la Terrasse, 91190 Gif-sur-Yvette, France; sana.slimene22@gmail.com (S.S.); michelle.david@i2bc.paris-saclay.fr (M.D.); 2 Département Recherche, Institut Curie, CNRS UMR3666, INSERM U 1339, 75005 Paris, France; daniel.dauzonne@orange.fr; 3 Molécules de Communication et Adaptation des Microorganismes (MCAM), Dept RDDM, UMR 7245 CNRS-MNHN, Muséum National d’Histoire Naturelle, CP54, 75005 Paris, France; renato.bensasson@gmail.com; 4 Institut de Chimie Physique, CNRS UMR 8000, Université Paris Saclay, 91405 Orsay, France

**Keywords:** Streptomyces, polyketide antibiotics, oxidative stress, nitro-flavone

## Abstract

Streptomyces *coelicolor* M145 (SC) is a model strain characterised by its highly active oxidative metabolism. This results in high levels of oxidative stress and the production of actinorhodin (ACT), a blue polyketide pigment with antioxidant properties. To determine whether oxidative stress triggers ACT biosynthesis in SC, we assessed the effect of adding five nitro-flavone (NF) derivatives to the growth medium. As NF molecules are well-known antioxidants, we hypothesized that their presence in the medium would moderate ACT production. The antioxidant efficiency of NF derivatives depends on their ability to donate electrons. This ability is linked to the nature of their 4’-substituent. The Quantitative Structure–Activity Relationship (QSAR) approach and Hammett constants predict the following order: OH > OCH_3_ > F > H > NO_2_. The results obtained demonstrate a clear dose–response relationship between the reduction in ACT production by SC and the antioxidant efficiency of these molecules. Therefore, these results strongly suggest that oxidative stress plays a pivotal role in initiating ACT biosynthesis. Furthermore, these results suggest that the direct spectrophotometric assay of ACT production by SC is a rapid, reliable, and inexpensive method of evaluating the in vivo antioxidant activity of any molecule, as predicted by the QSAR approach.

## 1. Introduction

**Figure 1:**
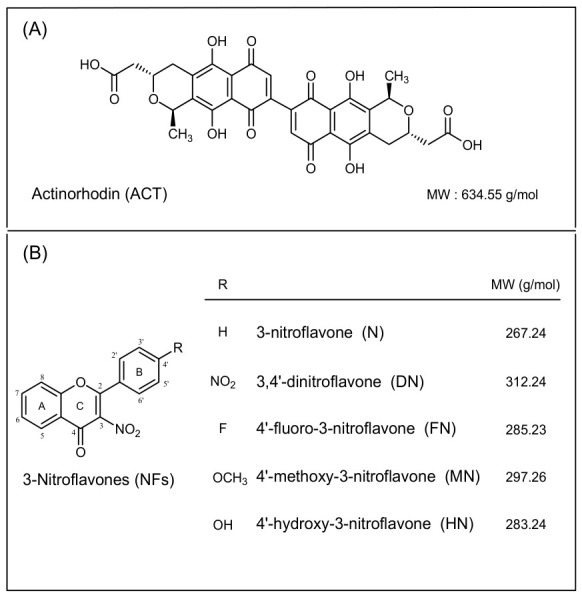
(**A**) Actinorhodin structure; (**B**) Nitroflavones structures with full names of the five 3-Nitroflavone derivatives and their molecular weights (MW). All chemical structures were drawn using ChemDraw Pro 8.0 (PerkinElmer Informatics, Cambridge, MA, USA).

Many bioactive molecules belonging to the polyketide family possess one or more quinone groups, giving them redox-active properties and enabling them to act as electron shuttles by capturing and donating electrons [1,2]. The extensively studied model strain Streptomyces *coelicolor* (SC) [3], for example, produces the blue benzoisochromane quinone molecule, actinorhodin (ACT) (Figure 1A) [4,5]. A strain derived from SC by deleting the M1141 ACT cluster was shown to be more sensitive to the thiol oxidant diamine than the original strain. This indicates that ACT has antioxidant properties [6].

Interestingly, other polyketides produced by actinomycetes [7,8] or fungi [9] such as chromomycin [10], collinolactone [11], natamycin/pimaricin [12,13] and tacrolimus [14] produced by *Streptomyces* species and lovastatin, penicillin and cephalosporin produced by fungal species [15,16] were also shown to play a role in the resistance to oxidative stress (OxS). For example, mutants of S. *natalensis* and S. *tsukubaensis* deleted for the AhpC gene (which encodes an enzyme that detoxifies H_2_O_2_) overproduce pimaricin and tacrolimus, respectively. This indicates that the accumulation of H_2_O_2_, has a positive impact on the biosynthesis of these specialised metabolites [14]. The biosynthesis of these antioxidant molecules is an adaptive response to OxS and is therefore expected to be induced by it. However, this has never been formally demonstrated in the case of ACT.

The aim of the present study was therefore to determine whether ACT biosynthesis was induced by OxS in SC. SC is characterised by highly activated oxidative metabolism, generating high levels of intracellular OxS [17,18,19]. This may explain the strong induction of ACT biosynthesis observed in this strain. To test this hypothesis, rather than increasing further the already high internal OxS of the SC strain by adding oxidative molecules to the culture medium [6], we preferred to reduce the high internal OxS of this strain by adding antioxidant molecules of the nitroflavone family [20] to its growth medium. To this end, the impact of three different concentrations of five 3-nitroflavone analogues (NFs) on ACT production was assessed. These five NFs were nitroflavone (N), 3,4′-dinitroflavone (DN), 4′-fluoro-3-nitroflavone (FN), 4′-methoxy-3-nitroflavone (MN) and 4′-hydroxy-3-nitroflavone (HN) (Figure 1B).

NFs were chosen for this test because the antioxidant properties of the selected NF derivatives have been clearly demonstrated in several in vitro and in vivo studies using different experimental models [21,22,23,24,25]. The hierarchy of efficacy of these compounds as antioxidants is related to their ability to donate electrons as predicted by the Quantitative Structure–Activity Relationship (QSAR) approach [26,27] and reflected in their Hammett constants (Table 1) [28,29]. The antioxidant ability of these molecules is notably related to the nature of their 4′-substituent, in the following order: OH (HN) > OCH3 (MN) > F (FN) > H (N) > NO2 (DN) (Figure 1B).

## 2. Materials and Methods

### 2.1. Preparation of Stock Solutions of the Five 3-Nitroflavones (NFs)

The five 3-nitroflavones used in this study were synthesized according to previously described procedures [30,31]. Stock solutions of the NFs were prepared by accurately weighing and dissolving approximately 10 to 20 mg of each NF in 1 mL of dimethyl sulfoxide (DMSO), the most biocompatible solvent, giving final concentrations ranging from 37 to 69 mM.L^−1^ (RSD = 2%), depending on the product. The final concentrations of each NF are stated in the figures and range from a minimum of 205 µM.L^−1^ to a maximum of 1296 µM.L^−1^, depending on the product.

### 2.2. Strain, Culture Conditions, and Assessment of Dry Biomass Yields and Intracellular ACT Production

This study uses the extensively researched model strain SC M145, which produces large quantities of ACT [5,32]. For all experiments, 10^6^ spores of this strain were plated onto a permeable, porous cellophane disc (Focus Packaging & Design Ltd., Louth, UK) and placed on the surface of 5 cm diameter agar plates containing 8 mL of modified R2YE agar medium with limited phosphate (no addition of K_2_HPO_4_) and no sucrose [33]. The cellophane disc makes it easy to scrape off the mycelium, enabling the dry biomass yield and intracellular ACT production to be determined. Small volumes of DMSO and H2O (used as controls), and of the NFs stock solutions (50 µL, 100 µL and 150 µL) were added to the agar plates containing 8 mL of R2YE culture medium [33], in order to assess the potential toxicity of DMSO and the NFs on bacterial growth and intracellular ACT production. Each plating was performed in quadruplicate to assess biomass yield and in triplicate to assess ACT production. After 48 h of incubation at 20 °C in the dark, the biomass yield and intracellular ACT production were evaluated for each plate. The mycelium from each plate was scraped from the cellophane membrane with a spatula and the dry biomass was weighed following lyophilization. The mean dry biomass was then calculated and presented as a histogram, with error bars showing the standard error.

Fifty milligrams of dry biomass were used to extract ACT. The biomass was incubated in 2 mL of 1.0 M KOH at 4 °C with constant agitation for two hours. After centrifugation, the supernatant was collected and this process was repeated once. The combined supernatants (4 mL) were mixed with 1 mL of 4.0 M HCl (final concentration of 1 M) to precipitate the ACT. The resulting precipitate was collected by centrifugation and resuspended in 1.0 M KOH. The optical density of the solution was then determined at 640 nm using a Shimadzu UV-1800 spectrophotometer (Shimadzu Corporation, Noisiel, France), with 1.0 M KOH used as the blank. The amount of ACT produced was calculated according to its molar absorbance at 640 nm (ε = 25,320 L mol^−1^ cm^−1^) and expressed in nanomoles per mg of dry biomass [33].

We used the Turkey test [34] to compare means, with *n* = 4 for biomass yield and *n* = 3 for ACT production. *p* values greater than 0.05 were considered non-significant.

## 3. Results

### 3.1. Effect of Adding DMSO and the Five 3-Nitroflavone Analogues on Biomass Yield

Nitroflavones (NFs) are practically insoluble in water, but fully soluble in dimethyl sulfoxide (DMSO). DMSO is the only biocompatible solvent that can be used to incorporate NFs into the culture medium of Streptomyces coelicolor (SC). To test the toxicity of DMSO, 50 µL, 100 µL and 150 µL of the solvent were added to the SC culture medium, with an equivalent volume of water added as a control.

**Figure 2:**
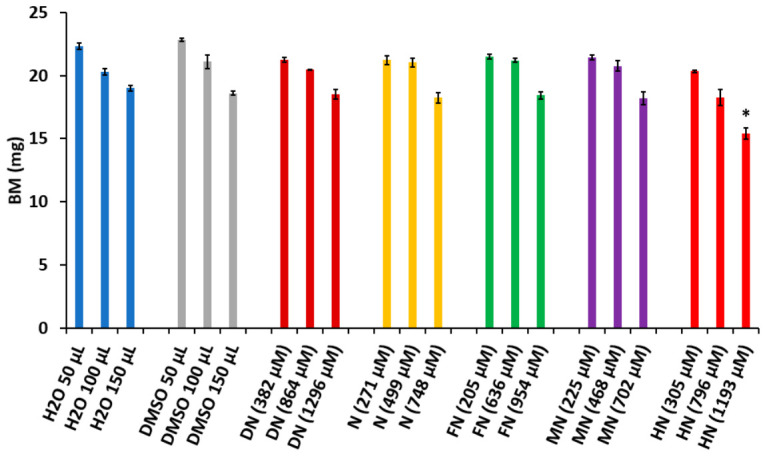
Histograms representing the effects of the addition of 50, 100, and 150 µL of H_2_O (blue histograms), DMSO (grey histograms) as well as of stock solutions of the five 3-Nitroflavone derivatives (NFs) solubilized in DMSO, on biomass (BM) yield of Streptomyces *coelicolor* M145 grown on 5 cm diameter agar plates containing 8 mL of the classical R2YE medium. Plates were incubated for 48 h at 20 °C in obscurity. The NFs tested are DN/3.4′-Dinitroflavone (brown histograms); N/3-Nitroflavone (yellow histograms); FN/4′-Fluoro-3-nitroflavone (green histograms); MN/4′-Methoxy-3-nitroflavone (purple histograms) and HN/4′-Hydroxy-3-nitroflavone (red histograms). The final concentration of each NF is stated under each histogram. The mean values are shown as histograms, with error bars indicating the standard deviation of the mean biomass yield across four independent cultures. * Tukey-adjusted comparisons reveal that the difference is significant (*p* < 0.05) for HN (1193 µM).

Figure 2 showed that adding 150 µL of dimethyl sulfoxide (DMSO) to the culture medium slightly reduced biomass yield, but this difference was not significant. As the concentrations of DMSO used were not toxic to the bacteria, we investigated the impact of adding 50 µL, 100 µL, or 150 µL of stock solutions from four of the five NFs to the culture medium on BM yield in milligrams. Adding 150 µL of the stock solutions from four of the five NFs had no significant negative effect on BM yield. However, adding 150 µL of HN resulted in a slight but significant decrease in BM yield. This suggests that HN is slightly toxic to the bacteria at a high concentration of 1193 µM/L, whereas DN, applied at an even higher concentration of 1296 µM/L, is not toxic.

### 3.2. Effect of Adding DMSO and the Five 3-Nitroflavones on Intracellular ACT Production

First, we visually inspected the plates after incubating them for 48 h at 20 °C in the dark, in order to evaluate the impact of adding 100 µL of H_2_O, DMSO, and medium concentrations of NFs on ACT production. The disappearance of the blue colour of the mycelium was clearly observed at medium concentrations ranging from 468 to 864 µM, indicating that the presence of NFs inhibited ACT production.

**Figure 3:**
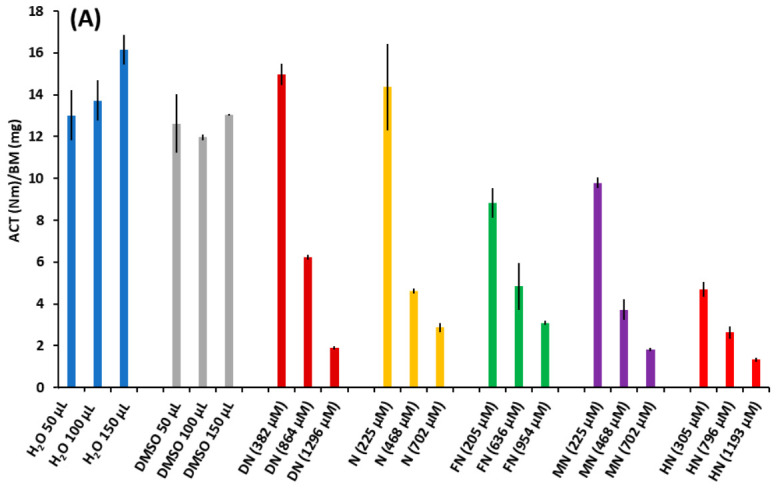

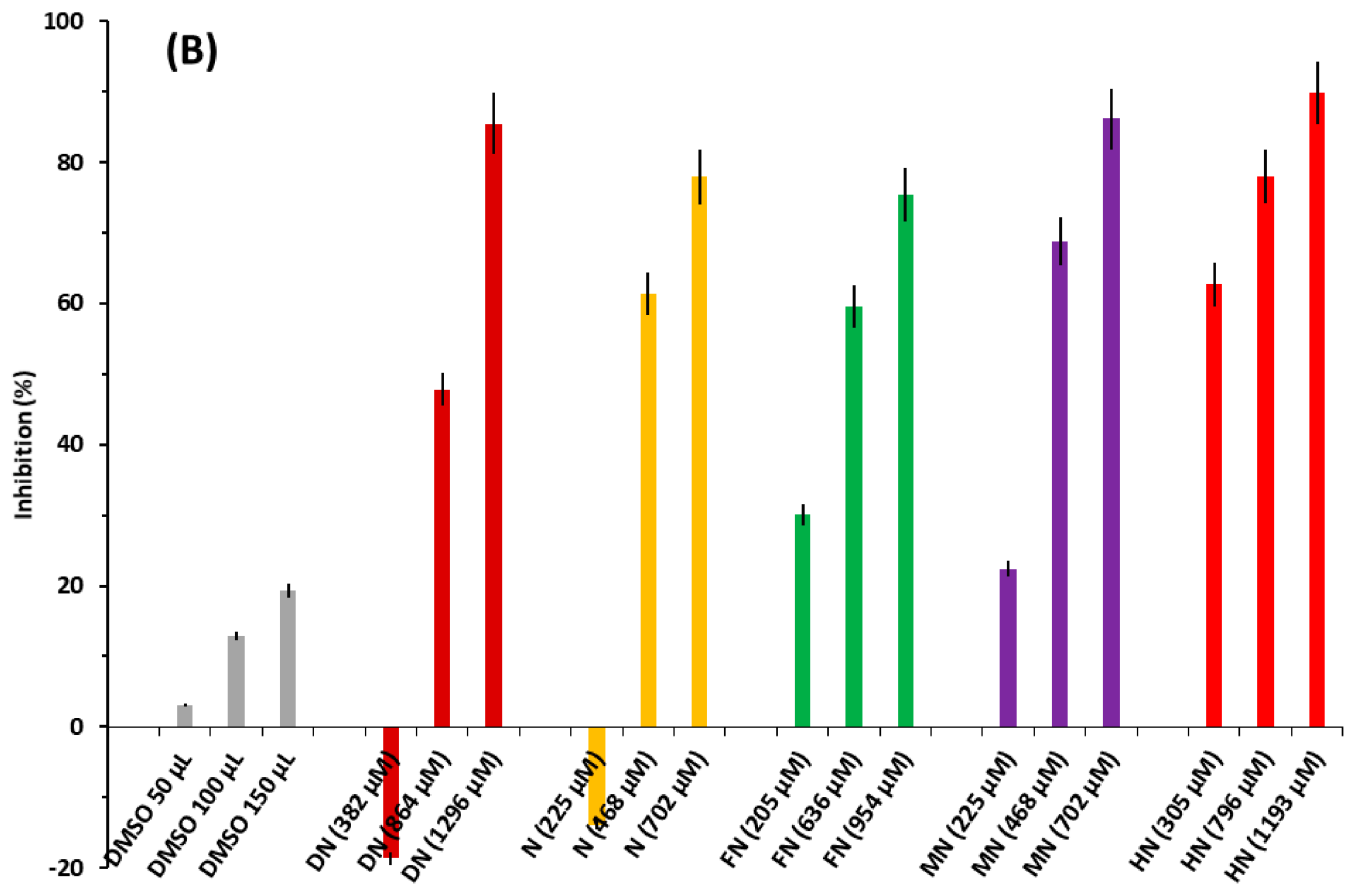
Histograms representing the concentration-dependent effect of the 3-nitroflavone derivatives (NFs) on the intracellular production of actinorhodin (ACT) of S. *coelicolor* M145. (**A**) ACT production expressed in nM/mg of dry biomass (BM) as a function of different concentrations of the five different NFs. (**B**) Percentage of inhibition of ACT production as a function of NF concentration. The growth conditions and the color code used for each NF are the same as in Figure 2. The final concentration of each NF is stated under each histogram. The mean values are shown as histograms, with error bars indicating the standard deviation in ACT production across three independent cultures.

To confirm these observations, we quantified the impact of three different concentrations of the five NFs on ACT production (Figure 3A). Figure 3A illustrates the various levels of intracellular ACT production in the presence of H_2_O, DMSO, and the five NFs in DMSO. DMSO appears to have a slight inhibitory effect on ACT production compared to water. However, a significant decrease in ACT production was observed for all NFs at medium and high concentrations compared to the water and DMSO controls. We then expressed these results as a percentage inhibition of ACT production (Figure 3B).

The percentage inhibition was calculated for each volume of DMSO (50, 100 and 150 µL) used, in comparison with the same volumes of H_2_O. DMSO was found to have a low, yet significant, volume-dependent inhibitory effect on ACT production compared to water at the same volumes (Figure 3B), but it had no effect on bacterial growth (Figure 2). This effect is consistent with the known antioxidant properties of DMSO [35] and also incidentally supports our hypothesis that oxidative stress plays a role in the triggering of ACT production.

In order to consider the antioxidant effect of DMSO, we calculated the % inhibition of the different NFs, relative to the volume of DMSO added (50, 100, or 150 µL). As can be seen in Figure 3B, the percentage inhibition increases in proportion to the concentration of NF added to the medium. However, we observed that the inhibitory effect of the lowest concentrations of DN and N, with a solvent volume of 50 µL of DMSO, was weaker than that of DMSO alone at the same volume. This resulted in inhibition percentages of −18% and −13%, respectively. This slight but significant antagonistic effect is intriguing and difficult to interpret, given that an additive or even synergistic effect was anticipated since DMSO also exhibits antioxidant properties. This effect was only observed at low doses of the two NFs. Further research is needed to confirm these findings. Taken together, however, these results clearly demonstrate that adding an antioxidant to the culture medium inhibits the production of ACT in SCs. This suggests that ACT production is dependent on OxS.

### 3.3. Dose–Response Relationship Between NF Concentration and ACT Production and Comparison of the Inhibitory Effect of the Five NFs on ACT Production

**Figure 4:**
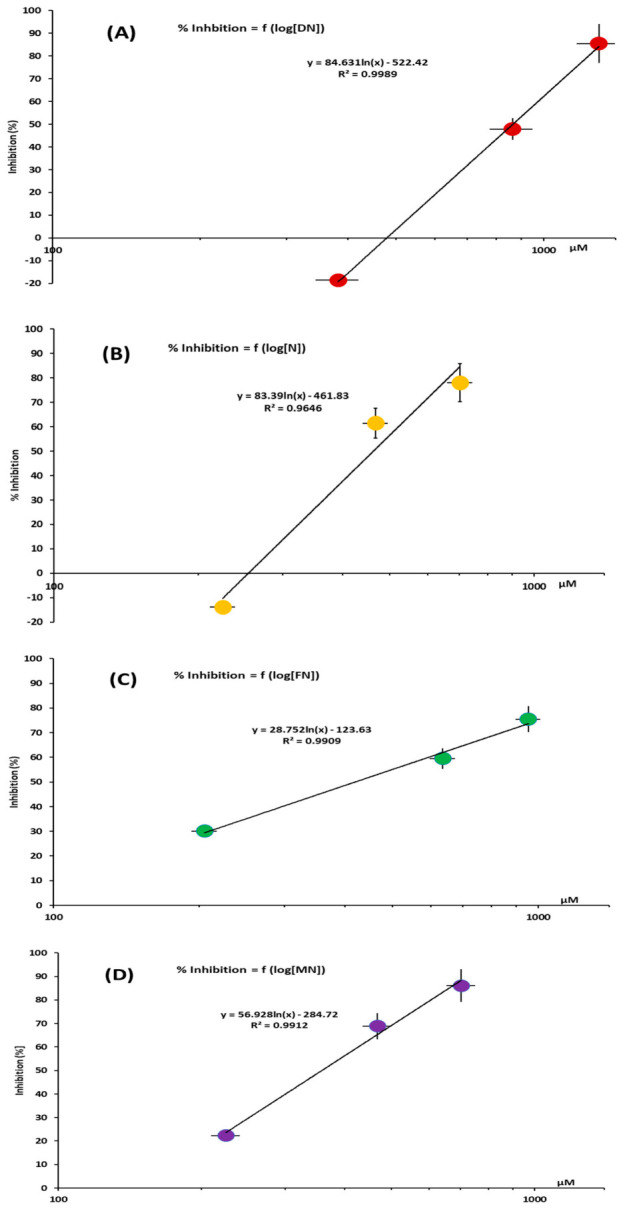

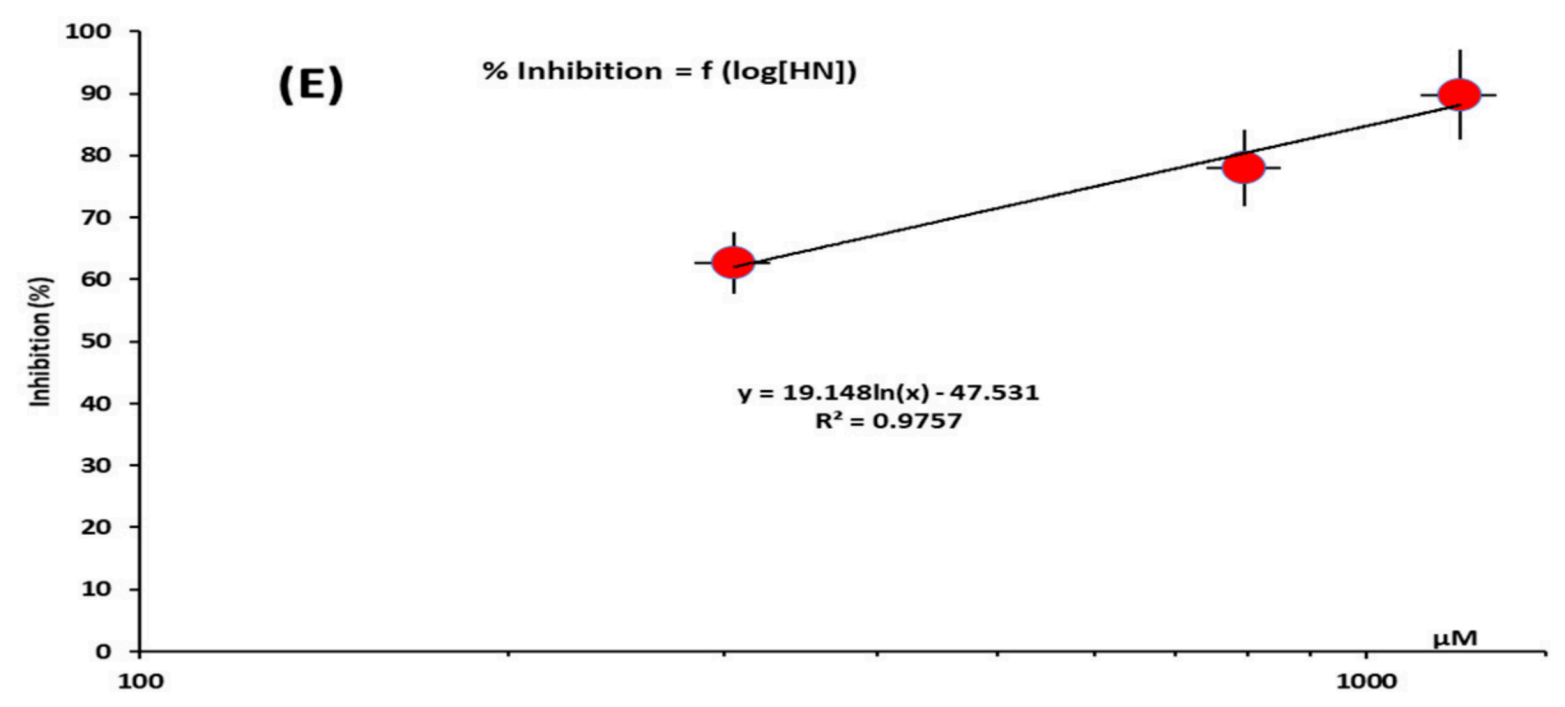
Linear correlations observed between the % inhibition of ACT production and the logarithm of the concentration of NFs in the culture medium (semi-logarithmic scale). (**A**) DN/3,4′-Dinitroflavone; (**B**) N/3-Nitroflavone; (**C**) FN/4′Fluoro-3-nitroflavone; (**D**) MN/4′Methoxy-3-nitroflavone; (**E**) HN/4′-Hydroxy-3-nitroflavone.

To compare the inhibitory effects of the different NFs tested, we determined the concentration that inhibits 50% of ACT production (the IC_50_). To achieve this, we plotted the percentage inhibition against the concentration of each NF in the culture medium on a semi-logarithmic scale (Figure 4A–E).

Figure 4 clearly shows that there is a linear dose–response relationship between the different concentrations of the five NFs present in the growth medium and the % inhibition of intracellular ACT production with a satisfactory coefficient of determination (R^2^) greater than 0.96 in all instances. This indicates that the dose–response curve is classically sigmoid on a normal scale, as expected. Therefore, the graphs in Figure 4 can be used to determine the IC_50_ value of each NF graphically (Table 1).

However, the IC_50_ value of HN cannot be accurately determined as it is below the lowest tested concentration. Nevertheless, its value is significantly lower than that of the other molecules, indicating that HN is the most effective derivative.

Figure 3 and Figure 4, as well as Table 1, show that 4′-hydroxy-3-nitroflavone (HN) exhibits the most significant inhibitory effect on ACT synthesis. To check whether the in vivo inhibitory effect of the five NFs on ACT production was related to their antioxidant abilities, as predicted by the QSAR approach [24,28,36,37,38,39] and reflected in their Hammett constants [27], we examined the correlation between the NFs’ inhibitory effects on ACT production and their Hammett constants.

### 3.4. Correlation Between Hammett’s Constants of the NFs and Their Ability to Inhibit ACT Production

The QSAR approach enables the antioxidant abilities of the NF derivatives used in this study to be ranked according to their ability to donate electrons. The strength of this ability is related to the nature of their 4′ substituent, in the following order: OH (HN) > OCH_3_ (MN) > F (FN) > H (N) > NO_2_ (DN). This is reflected in their Hammett constants (Table 1) [24,27,28]. To check the consistency between the antioxidant abilities of these molecules, as calculated by the QSAR approach and as determined by our in vivo experimental approach, we plotted the IC_50_ of the five NF derivatives as a function of their Hammett constants.

**Figure 5:**
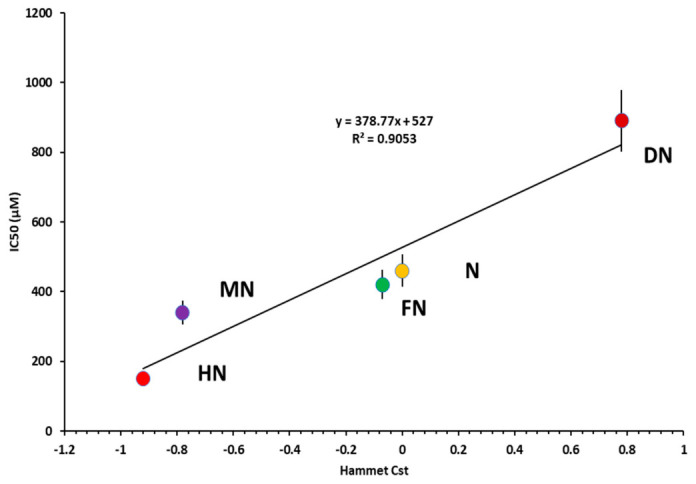
Linear correlation observed between the % inhibition of ACT production by the NFs, expressed as IC_50_, and their Hammet’s constants. DN/3,4′-Dinitroflavone (brown dots); N/3-Nitroflavone (yellow dots); FN/4′Fluoro-3-nitroflavone (green dots); MN/4′-Methoxy-3-nitroflavone (purple dots) and HN/4′Hydroxy-3-nitroflavone (red dots). The equation of the linear correlation is provided in the figure.

Figure 5 shows a good correlation (R^2^ > 0.90) between the inhibition of ACT production and the Hammett constants of the five NFs. This finding is consistent with previous data obtained using the QSAR approach obtained with other in vitro and in vivo experimental models used to determine the antioxidant abilities of various molecules [20,21,22,23,24].

Interestingly, these results suggest that the production of ACT by Streptomyces coelicolor M145 could serve as a rapid, reliable and inexpensive experimental model for testing the in vivo antioxidant properties of molecules predicted/calculated by the QSAR approach.

## 4. Discussion

Overall, our results demonstrate that adding NFs with well-established antioxidant properties to the SC culture medium significantly reduces ACT biosynthesis in a dose- and NF-type-dependent manner. The extent of this decrease varies depending on the ability of each NF to donate electrons. Although we did not measure intracellular reactive oxygen species (ROS) levels, the fact that well-known antioxidants such as NFs decrease ACT biosynthesis clearly shows that OxS is essential for ACT biosynthesis in SC. Indeed, this strain is characterized by a strongly activated oxidative metabolism, generating high levels of OxS. This is likely to be responsible for the strong induction of ACT biosynthesis [16,17,18]. In this respect, it is interesting to note the structural similarities between the ACT (Figure 1A) and the NFs (Figure 1B), as both contain phenolic/aromatic cycles with quinone groups that are known to impart redox-active properties to these molecules [40]. These molecules can act as electron shuttles, capturing and donating electrons. Their structural similarities are consistent with their antioxidant function [40]. However, in order to clearly establish the mechanism of action of NFs, it is necessary to investigate the intermediate regulatory steps linking NF exposure to reduced ACT production. This should involve measuring levels of intracellular ROS, oxidative-stress markers, and the expression of pathway-specific regulatory genes, including actII-ORF4 [41].

Numerous in vivo studies indicate that these small molecules can cross cell walls and lipid membranes, acting intracellularly as antioxidants inside cells to reduce OxS. Although such behaviour has been reported in other experimental systems, and although the results obtained strongly suggest that NFs enter the cells, this study is limited in that it does not provide direct evidence of uptake or intracellular activity of NFs in S.C. To address this limitation, it is essential to determine the concentration of NFs in the dry mycelium. This work is currently in progress.

Interestingly, the negative effect that NFs exert on ACT production was found to be linearly correlated with the antioxidant efficacy hierarchy of NFs. This hierarchy is linked to the ability of NFs to donate electrons, as predicted by the QSAR approach. Consequently, the simple spectrophotometric assay used to quantify the amount of ACT produced by SC provides a quick, reliable and inexpensive biotechnological method of assessing the in vivo antioxidant activity of small molecules, as predicted by the QSAR approach.

However, it should be noted that knowledge of the regulatory cascades linking OxS to the expression of specific specialised metabolite biosynthetic pathways in Streptomyces is limited. Only two recent studies have reported that the biosynthesis of ACT in SC is positively regulated by reactive nitrogen species generated endogenously, such as nitrate (NO_3_^−^), nitrite (NO_2_^−^) and nitric oxide (NO) [42]. In fact, SC mutants with a reduced ability to produce NO exhibit significantly lower levels of ACT production. This process is regulated by the DevS/DevR two-component system [42,43]. DevS is a haem-containing kinase that detects intracellular NO [42], whereas DevR is a response regulator that is phosphorylated by DevS in an NO level-dependent manner [43]. Phosphorylated DevR is able to directly interact with the promoter region of actII-ORF4, which encodes the activator of the ACT cluster, thereby activating its expression, as well as that of the ACT cluster itself [41]. Another study suggests a link between ACT and OxS. Indeed, ACT has been shown to induce the expression of a small regulon of genes under the positive control of the SoxR/SCO1697 regulator, which likely carries [2Fe-2S] clusters that sense OxS [44,45]. The SoxR regulon comprises 5 genes encoding an ABC transporter (SCO7008), an NAD-dependent epimerase/dehydratase (SCO1178) as well as two probable NADPH-dependent oxidoreductases (SCO2478 and SCO4266); and a monooxygenase (SCO1909) [46].

The function of the enzymes of the SoxR regulon is still not clearly elucidated. Some authors have proposed that they may be involved in the efflux and detoxification of ACT [44]. However, based on our study, we hypothesize that some of these enzymes catalyze the oxidoreduction of ACT in order to restore its antioxidant/reducing capacity. Another study demonstrated that OxS plays a role in regulating the expression of the natamycin/pimaricin biosynthetic pathway, which is another specialized metabolite of the polyketide family produced by S. gilvosporeus [13]. In this strain, the OxyR regulator positively controls the expression of SgnM, which encodes a specific activator situated in the natamycin biosynthetic pathway [13]. The Cys212 and Cys221 residues of the H_2_O_2_-sensing domain of OxyR undergo oxidation in response to ROS, which are generated by overproducing of cholesterol oxidase, a catalyst of sterol oxidation that produces H_2_O_2_. This triggers a conformational change that enables OxyR to interact with specific motifs in the promoter region of sgnM [13]. This redox-dependent process controls the transcription of the sgnM gene, thereby regulating the expression of the 12 genes in the natamycin/pimaricin pathway.

Therefore, we can conclude that the polyketide family of specialised metabolites, which all possess a quinone group, are predicted to exhibit antioxidant properties. Furthermore, we can hypothesise that the activation of these pathways in response to OxS is a common regulatory feature.

In summary, further work is required to fully elucidate the mechanism by which NFs reduce ACT production. Direct measurements of intracellular ROS levels, oxidative-stress markers and key regulatory genes expression, such as actII-ORF4, would also considerably strengthen the proposed model.

It is also worth noting that a recent study [47] describes how certain flavonoids induce the formation of wall-deficient cells in SC. This provides relevant precedent in support of the idea that flavonoids may influence SC development and physiology.

## Figures and Tables

**Table 1:** Nitroflavones’ inhibitory concentrations 50 (IC50), as determined under our operating conditions (RSD < 10%), and Hammet constants [27]. * Although the IC50 value of HN is inaccurate, it is clearly much lower than those of the other molecules.

NF	IC50 (µM)	RSD (n = 3)	Hammet Cst
DN	890	10	0.78
N	460	10	0
FN	420	7	−0.07
MN	340	8	−0.78
HN	150 *	8	−0.92

## Data Availability

The original contributions presented in this study are included in the article. Further inquiries can be directed to the corresponding authors.

## Abbreviations

The following abbreviations are used in this manuscript:

OxS	oxidative stress
SC	Streptomyces coelicolor M145
ACT	Actinorhodin
NF	Nitro-flavone
QSAR	Quantitative Structure–Activity Relationship

## References

[1] Lu J.M., Rosokha S.V., Neretin I.S., Kochi J.K. (2006). Quinones as electron acceptors. X-ray structures, spectral (EPR, UV-vis) characteristics and electron-transfer reactivities of their reduced anion radicals as separated vs contact ion pairs. J. Am. Chem. Soc..

[2] Franza T., Gaudu P. (2022). Quinones: More than electron shuttles. Res. Microbiol..

[3] Bentley S.D., Chater K.F., Cerdeño-Tárraga A.-M., Challis G.L., Thomson N.R., James K.D., Harris D.E., Quail M.A., Kieser H., Harper D. (2002). Complete genome sequence of the model actinomycete *Streptomyces coelicolor* A3(2). Nature.

[4] Malpartida F., Hopwood D.A. (1984). Molecular cloning of the whole biosynthetic pathway of a Streptomyces antibiotic and its expression in a heterologous host. Nature.

[5] Bystrykh L.V., Fernández-Moreno M.A., Herrema J.K., Malpartida F., Hopwood D.A., Dijkhuizen L. (1996). Production of actinorhodin-related “blue pigments” by *Streptomyces coelicolor* A3(2). J. Bacteriol..

[6] Millan-Oropeza A., Henry C., Lejeune C., David M., Virolle M.J. (2020). Expression of genes of the Pho regulon is altered in *Streptomyces coelicolor*. Sci. Rep..

[7] Yu H.Q., Li G., Lou H.X. (2022). Isolation, Biosynthesis, and Biological Activity of Polycyclic Xanthones From Actinomycetes. Front. Microbiol..

[8] Risdian C., Mozef T., Wink J. (2019). Biosynthesis of Polyketides in Streptomyces. Microorganisms.

[9] Christiansen J.V., Isbrandt T., Petersen C., Sondergaard T.E., Nielsen M.R., Pedersen T.B., Sørensen J.L., Larsen T.O., Frisvad J.C. (2021). Fungal quinones: Diversity, producers, and applications of quinones from Aspergillus, Penicillium, Talaromyces, Fusarium, and Arthrinium. Appl. Microbiol. Biotechnol..

[10] Prajapati D., Kumari N., Dave K., Chatupale V., Pohnerkar J. (2018). Chromomycin, an antibiotic produced by *Streptomyces flaviscleroticus* might play a role in the resistance to oxidative stress and is essential for viability in stationary phase. Environ. Microbiol..

[11] Schmid J.C., Frey K., Scheiner M., Garzón J.F.G., Stafforst L., Fricke J., Schuppe M., Schiewe H., Zeeck A., Weber T. (2021). The Structure of Cyclodecatriene Collinolactone, its Biosynthesis, and Semisynthetic Analogues: Effects of Monoastral Phenotype and Protection from Intracellular Oxidative Stress. Angew. Chem. Int. Ed. Engl..

[12] Beites T., Pires S.D.S., Santos C.L., Osório H., Moradas-Ferreira P., Mendes M.V. (2011). Crosstalk between ROS homeostasis and secondary metabolism in *S. natalensis* ATCC 27448: Modulation of pimaricin production by intracellular ROS. PLoS ONE.

[13] Zong G., Cao G., Fu J., Zhang P., Chen X., Yan W., Xin L., Wang Z., Xu Y., Zhang R. (2023). Novel mechanism of hydrogen peroxide for promoting efficient natamycin synthesis in Streptomyces. Microbiol. Spectr..

[14] Pires S.D.S., Oliveira R., Moradas-Ferreira P., Mendes M.V. (2020). The Onset of Tacrolimus Biosynthesis in *Streptomyces tsukubaensis* Is Dependent on the Intracellular Redox Status. Antibiotics.

[15] Miranda R.U., Gómez-Quiroz L.E., Mendoza M., Pérez-Sánchez A., Fierro F., Barrios-González J. (2014). Reactive oxygen species regulate lovastatin biosynthesis in *Aspergillus terreus* during submerged and solid-state fermentations. Fungal Biol..

[16] Bibian M.E., Perez-Sanchez A., Mejia A., Barrios-Gonzalez J. (2020). Penicillin and cephalosporin biosyntheses are also regulated by reactive oxygen species. Appl. Microbiol. Biotechnol..

[17] Esnault C., Dulermo T., Smirnov A., Askora A., David M., Deniset-Besseau A., Holland I.-B., Virolle M.-J. (2017). Strong antibiotic production is correlated with highly active oxidative metabolism in *Streptomyces coelicolor* M145. Sci. Rep..

[18] Millan-Oropeza A., Henry C., Blein-Nicolas M., Aubert-Frambourg A., Moussa F., Bleton J., Virolle M.-J. (2017). Quantitative Proteomics Analysis Confirmed Oxidative Metabolism Predominates in *Streptomyces coelicolor* versus Glycolytic Metabolism in *Streptomyces lividans*. J. Proteome Res..

[19] Lejeune C., Sago L., Cornu D., Redeker V., Virolle M.J. (2022). A Proteomic Analysis Indicates That Oxidative Stress Is the Common Feature Triggering Antibiotic Production in *Streptomyces coelicolor* and in the *pptA* Mutant of *Streptomyces lividans*. Front. Microbiol..

[20] Banjarnahor S.D.S., Artanti N. (2015). Antioxidant Properties of Flavonoids. Med. J. Indones..

[21] Steele V.E., Boone C.W., Dauzonne D., Rao C.V., Bensasson R.V. (2002). Correlation between electron-donating ability of a series of 3-nitroflavones and their efficacy to inhibit the onset and progression of aberrant crypt foci in the rat colon. Cancer Res..

[22] Hassanpour S.H., Doroudi A. (2023). Review of the antioxidant potential of flavonoids as a subgroup of polyphenols and partial substitute for synthetic antioxidants. Avicenna J. Phytomed..

[23] Pejcic T., Zeković M., Bumbaširević U., Kalaba M., Vovk I., Bensa M., Popović L., Tešić Ž. (2023). The Role of Isoflavones in the Prevention of Breast Cancer and Prostate Cancer. Antioxidants.

[24] Scurlock R., Rougee M., Bensasson R.V. (1990). Redox properties of phenols, their relationships to singlet oxygen quenching and to their inhibitory effects on benzo(a)pyrene-induced neoplasia. Free Radic. Res. Commun..

[25] Bensasson R.V., Hashjin S.S., Zoete V., Dauzonne D., Matta C.F. (2013). Physicochemical properties of exogenous molecules correlated with their biological efficacy as protectors against carcinogenesis and inflammation. Int. Rev. Phys. Chem..

[26] Hansch C., Hoekman D., Leo A., Zhang L., Li P. (1995). The expanding role of quantitative structure-activity relationships (QSAR) in toxicology. Toxicol. Lett..

[27] Hansch C., Zhang L. (1995). Comparative QSAR: Radical toxicity and scavenging. Two different sides of the same coin. SAR QSAR Environ. Res..

[28] Hammett L.P. (1937). The Effect of Structure upon the Reactions of Organic Compounds. Benzene Derivatives. J. Am. Chem. Soc..

[29] Hansch C., Maloney P.P., Fujita T., Muir R.M. (1962). Correlation of Biological Activity of Phenoxyacetic Acids with Hammett Substituent Constants and Partition Coefficients. Nature.

[30] Dauzonne D., Grandjean C. (1992). Synthesis of 2-aryl-3-nitro-4H-1-benzopyran-4-ones. Synthesis.

[31] Dauzonne D., Folleas B., Martinez L., Chabot G.G. (1997). Synthesis and in vitro cytotoxicity of a series of 3-aminoflavones. Eur. J. Med. Chem..

[32] Marshall A.P., Carlson E.E. (2023). Metabolomics Reveals a “Trimeric” gamma-Actinorhodin from *Streptomyces coelicolor* M145. Chembiochem.

[33] Kieser T., Bibb M.J., Buttner M.J., Chater K.F., Hopwood D.A. (2000). Practical Streptomyces Genetics.

[34] National Institute of Standards and Technology (.gov) Tukey’s Method—Information Technology Laboratory. https://www.itl.nist.gov/div898/handbook/prc/section4/prc471.htm.

[35] Sanmartín-Suárez C., Soto-Otero R., Sánchez-Sellero I., Méndez-Álvarez E. (2011). Antioxidant properties of dimethyl sulfoxide and its viability as a solvent in the evaluation of neuroprotective antioxidants. J. Pharmacol. Toxicol. Methods.

[36] Mauri A., Consonni V., Todeschini R. (2017). Molecular Descriptors. Handbook of Computational Chemistry.

[37] Soares T.A., Nunes-Alves A., Mazzolari A., Ruggiu F., Wei G.-W., Merz K. (2022). The (Re)-Evolution of Quantitative Structure-Activity Relationship (QSAR) Studies Propelled by the Surge of Machine Learning Methods. J. Chem. Inf. Model..

[38] Roy K., Kar S., Das R.N. (2015). A Primer on QSAR/QSPR Modeling: Fundamental Concepts.

[39] Ghasemi F., Mehridehnavi A., Perez-Garrido A., Perez-Sanchez H. (2018). Neural network and deep-learning algorithms used in QSAR studies: Merits and drawbacks. Drug Discov. Today.

[40] Kristensen S.B., van Mourik T., Pedersen T.B., Sørensen J.L., Muff J. (2020). Simulation of electrochemical properties of naturally occurring quinones. Sci. Rep..

[41] Arias P., Fernández-Moreno M.A., Malpartida F. (1999). Characterization of the Pathway-Specific Positive Transcriptional Regulator for Actinorhodin Biosynthesis in *Streptomyces coelicolor* A3(2) as a DNA-Binding Protein. J. Bacteriol..

[42] Honma S., Ito S., Yajima S., Sasaki Y. (2021). Nitric Oxide Signaling for Actinorhodin Production in *Streptomyces coelicolor* A3(2) via the DevS/R Two-Component System. Appl. Environ. Microbiol..

[43] Honma S., Ito S., Yajima S., Sasaki Y. (2023). Role of DevR phosphorylation in nitric oxide homeostasis and signaling of *Streptomyces coelicolor* A3(2) M145. FEMS Microbiol. Lett..

[44] Dela Cruz R., Gao Y., Penumetcha S., Sheplock R., Weng K., Chander M. (2010). Expression of the *Streptomyces coelicolor* SoxR regulon is intimately linked with actinorhodin production. J. Bacteriol..

[45] Shin J.H., Singh A.K., Cheon D.J., Roe J.H. (2011). Activation of the SoxR regulon in *Streptomyces coelicolor* by the extracellular form of the pigmented antibiotic actinorhodin. J. Bacteriol..

[46] Naseer N., Shapiro J.A., Chander M. (2014). RNA-Seq analysis reveals a six-gene SoxR regulon in *Streptomyces coelicolor*. PLoS ONE.

[47] Valdés-Chiara P., Alonso-Fernández S., Manteca A., Fernández-García G. (2026). The Flavonoids Daidzein and Genistein Induce Wall-Deficient Cell Formation in *Streptomyces coelicolor* Under Hyperosmotic Stress. Microb. Biotechnol..

